# Necrotizing fasciitis in neonates: A case report and review of literature

**DOI:** 10.1002/ccr3.8158

**Published:** 2023-11-06

**Authors:** Roya Oboodi, Hamide Barzegar, Roozbeh Behzadi

**Affiliations:** ^1^ Neonatal Research Center Shiraz University of Medical Sciences Shiraz Iran; ^2^ Department of Pediatrics, School of Medicine Shiraz University of Medical Sciences Shiraz Iran

**Keywords:** infant, necrotizing fasciitis, newborn, sepsis

## Abstract

Early recognition and treatment of necrotizing fasciitis reduce mortality rate which is 24.1% among neonates. Antibiotics and debridement are common treatments. Hyperbaric oxygen and negative pressure wound healing show potential but need further investigation.

## Graphical abstract

Necrotizing fasciitis, a rarely fatal but rapidly progressing disease, is less recognized in neonates. Polymicrobial infections are the primary culprits. As per the literature and a specific case, common clinical indications include erythema, fever, swelling, lethargy, and irritability. Diagnosis is primarily clinical, and treatment necessitates immediate surgical exploration and antimicrobial therapy.

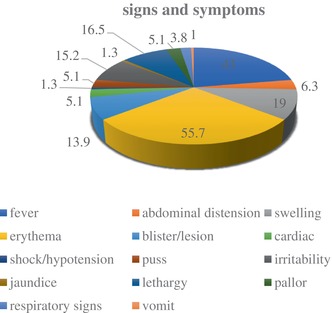

## 1 INTRODUCTION

Necrotizing fasciitis (NF) is a rare fatal rapidly progressive infection that destroys all skin layers and muscles.^1^ Due to nonspecific signs and symptoms, it may be misdiagnosed as cellulitis.^2^, ^3^ Prompt diagnosis and timely treatment are important factors in morbidity and mortality rates.^4^ The treatment includes broad‐spectrum antibiotic and surgical interventions.^5^ There is little information about NF in neonates; most published articles are case reports. We aimed to present a neonate with NF and then review articles in this age group with a focus on risk factors, signs and symptoms, microbial agents, treatment, and outcome.

## 2 CASE PRESENTATION

A 10‐day‐old girl presented with an erythematous lesion, with warmness and induration, in the abdomen. She was born at 34 weeks gestational age from a 31‐year‐old mother. The pregnancy was uneventful, except for intrauterine growth restriction (IUGR) and fetal distress which was the main reason for the preterm termination of the pregnancy with cesarean section. Her first‐ and fifth‐minute Apgar scores were 8 and 9, respectively. Growth indicators included a birth weight of 1250 g, a height of 43 cm, and head circumference of 28 cm. She was admitted to the neonatal intensive care unit due to prematurity, IUGR, and respiratory distress syndrome. She received oxygen for 1 day. Ampicillin and amikacin were started for her. After 3 days, when the blood culture was negative, we discontinued the antibiotics.

**FIGURE 1:**
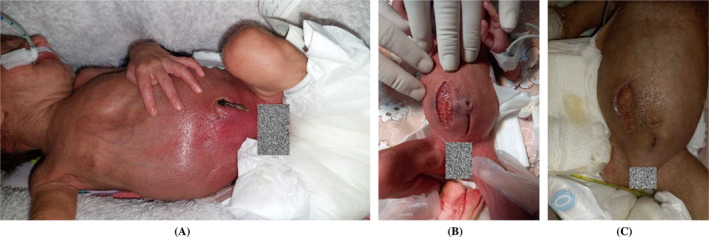
Evolution of necrotizing fasciitis lesion in a preterm neonate: (A) Redness, swelling, and rapid progression to necrosis (B) the discoloration around the umbilicus and two parallel incisions (C) the wound in the healing process.

On the 10th day, she presented with an erythematous lesion on the abdominal wall, the right side of the umbilicus, with no sign of omphalitis (Figure 1), which changed to necrosis rapidly. The patient had no umbilical line. Vancomycin (10 mg/kg/dose q12h), meropenem (20 mg/kg/dose q8h), and metronidazole (7.5 mg/kg/dose q24h) started for her, debridement, and irrigation were done via two parallel vertical incisions (Figure 1) with the diagnosis of necrotizing fasciitis continued by daily dressing with gentamycin.

The wound and blood culture were negative. On the 22nd day, she developed feeding intolerance, bilious drainage, and abdominal distension. As she had metabolic acidosis and severe restlessness, with the suspicion of volvulus or obstruction, an exploratory laparotomy was performed. She had normal small bowel, no sign of volvulus, and no thickening of the bowel loops. After 2 days, feeding started in a small amount. Another wound culture was sent that was Acinetobacter sensitive to colistin. Thus, we started colistin. She was in good condition, could tolerate feeding, and the healing site of incisions for NF was obvious. The next wound culture was negative. On the 27th day, she received packed red blood cell due to hematocrit 22.1 and need for oxygen. After that, she had no need to oxygen. On the 30th day, she became tachycardic (heart rate of 180) and on the next day, she developed tachypnea, retraction, hypotension, and lethargy. She was intubated. Pulmonary hemorrhage was obvious. Laboratory data were in favor of sepsis (C‐Reactive protein >100, WBC 1.6, Hb 8.8, PLT 70, neutrophil 33%). Unfortunately, she died.

## 3 DISCUSSION

As necrotizing fasciitis is rapidly progressing, delay in diagnosis and treatment is associated with severe morbidities and mortality. Here, we present a neonate who was a case of necrotizing fasciitis; then based on the search on Google Scholar and PubMed with the keywords, newborn, necrotizing fasciitis, and by excluding reports articles the full texts of which were not available, we collected 76 newborn cases and investigated their presentation, risk factor, treatment, and outcome in this age group (Table 1).

**TABLE 1:** Demographic and clinical characteristics of neonates with NF.

Study	Age (day)	GA	Sex	Site	Presentation	Risk factor	Treatment	Outcome	complication	Organism
Ameh et al.^6^	29	T		Scrotum	Reducible right inguinoscrotal hernia, poor feeding, irritability, abdominal distension, vomiting, fever		Debriding and closing the fascia and open skin Antibiotic	S	Post‐operation infection	
Kuroda et al.^5^, ^7^	27	T	M	Scrotum	Crying and swelling of left scrotum		Surgery cefmetazole and then amoxicillin)	S	Positive urine culture	s. agalactia
Barker et al.^5^, ^8^	4	T	M	Left flank area	Tachycardia Metabolic acidosis Discolored area in the left flank	Imperforate anus and operation	Debridement Antibiotics Daily manuka honey dressing		Positive blood and peritoneal culture	NF *E coli*
Inoue et al.^5^, ^9^	10	P	M	Abdomen	Erythema and ecchymosis	NEC	Arbekacin Cefmetazole	D	Sepsis DIC	Zygomycetes
Lebel et al.^5^, ^10^	21		F	Upper limb	Fever Restlessness Swelling and redness of right upper limb	Laying in the park	Cefazolin Vancomycin Cefotaxime Surgery	S	Positive blood culture Septic shock	Group A streptococcus
Melchionda et al.^5^, ^11^	20		F	perineal	Red violaceous nodules	AML Chemotherapy	Topical negative pressure therapy with silver foam dressing Broad spectrum antibiotics	S		*P. aeruginosa*
Thapa et al.^5^, ^12^	7		F	Left upper arm	Fever Swelling and redness of left upper arm	BCG vaccination	Vancomycin clindamycin debridement surgical honey dressing	S		MRSA
Walls et al.^5^, ^13^	9	T	M	umbilical	Poor feeding Vomiting Abdominal distension Scrotal swelling Periumbilical redness Folliculitis of head and trunk (MRSA)		Debridement Antibiotic	D	Seizure Pulmonary hemorrhage Multiorgan failure Multiple hemorrhages in the cerebellum	MRSA Multiple growth
Shi et al.^14^	25		M	Left leg			Ceftazidim	D		
Shi et al.^14^	28		M	Buttock, back			ceftazidim	D		
Peker et al.^15^	1	P	F	Lower back	Deterioration of respiration and Shock		Vancomycin Meropenem	D		MRSA
Özkan et al.^16^	14	T	M	perianal	Red, painful, cutaneous lesion		Surgical debridement Ampi sulbactam Ceftazidime	S		*Klebsiella pneumoniae*
Wu et al.^17^	5	T	M	Left back	Large area of bluish red discoloration Fever lethargy		Antibacterial treatment	S	Positive blood culture	MRSA
Negosanti et al.^18^	20	T	F	Perianal	Perianal erythema and hemorrhagic pustule	AML Chemotherapy	Meropenem Vancomycin Tobramycin Surgical debridement IVIG Advanced dressing (silver polyurethane foam and hydrofiber) Topical Negative pressure device	S		*Pseudomonas aeruginosa*
Narvey et al.^19^	23	P	F	Abdominal wall	Tachycardia Irritability Erythematous, indurated tender skin		Ampicillin Gentamycin Clindamycin Debridement	S	Positive blood culture (gram positive cocci)	GBS
Ganesh et al.,^20^, ^21^	14		M		Ulcer of medial cantus of left eye	Nasolacrimal obstruction LADs 1	Amikacin Metronidazole Meropenem	S	Hard palate perforation	*Pseudomonas aeruginosa*
Bliss Jr et al.^22^	10	T	M		Fever Erythema of genitalia and lower abdomen	Plastibell circumcision	Debridement Ampicillin Gentamicin Clindamycin Hyperbaric oxygen	S	Positive blood culture (coagulase‐negative *Staphylococcus*)	Klebsiella Coagulase‐negative *Staphylococcus*
Bliss Jr et al.^22^	3	T	M	Scrotum	Pustule on the scrotum	Plastibell circumcision	Ampicillin Clindamycin Gentamicin Debridement Hyperbaric oxygen	S		*Staphylococcus aureus* *Staphylococcus epidermidis* Gram‐negative rods
Bodemer et al.^23^	21	T	F		Lethargy Necrotic and inflammatory region on the left hemithorax, scapular, shoulder		Oxacillin Ticarcillin Amikacin Debridement Skin autograft	S		
Bodemer et al.^23^	14		F	Left hemithorax	Left hemithorax lesion Fever Lethargy		Penicillin Erythromycin Debridement	S		MRSA
Bodemer et al.^23^	15		F	Right mammary region	Lethargy Painful and tense breast enlargement		Penicillin Erythromycin Debridement	S		MSSA
Bodemer et al.^23^	12		F	Left mammary region	Painful breast enlargement		Penicillin Erythromycin Kanamycin Debridement	S		MSSA
Chao et al.^24^	13	T	F	Back	Fever Erythema on back		Antibiotic therapy Debridement Skin graft	S		*Staphylococcus aureus*
Chao et al.^24^	17	T	M	Back	Fever Back swelling and erythema		Antibiotic therapy Debridement	S		*Staphylococcus aureus*
Christensen et al.^25^	3	P	M	Perianal	Erythematous extending ring around anus	Mother: GBS + Rectal temperature	Ampicillin Gentamicin	D	DIC Metabolic acidosis	
Dehority et al.^26^	5			Lower back	Fever Irritability Poor feeding	Mothers breast milk culture: *Staphylococcus aureus*	Meropenem Vancomycin Fluconazole Debridement	S		*Staphylococcus aureus*
Ekingen et al.^27^	10	P		Perianal	Perianal hyperemia		Meropenem Teicoplanin Debridement Colostomy		Anal stenosis and stricture	Negative culture
Fustes‐Morales et al.^28^	10		M	Trunk		Myelomeningocele ruptured Abdominal surgery		D	Sepsis DIC	Gram‐positive cocci
Hayani et al.^29^	9		M	Chest	Fever Rupture lesion near the left nipple		Vancomycin Clindamycin Debridement	S		MRSA
Hsieh et al.^30^	13	T	F	Back	Fever Irritability Erythematous area on lower back		Vancomycin Gentamicin Metronidazole Debridement Skin graft	S	Positive blood culture	MRSA
Hsieh et al.^30^	16	T	F	Back Buttock	Inflammatory lesion over lower back and buttock		Vancomycin Gentamicin Metronidazole Debridement	S		MRSA
Hsieh et al.^30^	7	T	F	Back	Fever Poor feeding Large erythematous lesion in the lumbar lesion		Vancomycin Gentamicin Metronidazole Debridement	S		MRSA
Ignacio et al.^31^		P	M	Abdomen Back Perianal Buttock	Erythema and ecchymosis along the wound edge	NEC Abdominal surgery	Debridement Antibiotic GCSF	D	Sepsis	Enterobacter
Zuloaga‐Salcedo et al.^32^	3	T	F	Buttock	Erythematous patch on the back		Vancomycin Clindamycin Ceftriaxone Debridement Alginate dressing Negative pressure therapy	S	Positive blood culture (enterobacter cloac, coagulase‐negative *Staphylococcus*)	
Orii et al.^33^	12	P		Lower extremity	Discoloration bellow ankle	Site of central venous catheter	Panipenem Vancomycin	D	Positive blood culture Sepsis DIC	MRSA
Dunlop et al.^34^	11	T	M	Buttock	Erythema	Mother GBS	Vancomycin Clindamycin Debridement Dressing with Mepitel_ and Acticoat Skin autograft	S		MSSA
Casey et al.^35^	51	P	M	Abdominal wall	Blistering and sloughi 39 ng of the skin	NEC	Resection of abdominal wall Vancomycin Meropenem Fluconazole Penicillin Allograft skin Negative pressure wound therapy	D	NEC Peritoneal fluid culture Enterococcus, two species of *Escherichia coli*, *Klebsiella oxytoc*a and *Proteus mirabilis*	
Kimia et al.^36^	4	T	F	Scalp	Blister on scalp Tachycardia Hypotensive	Fetal scalp monitoring	Debridement Broad spectrum Antibiotic Negative pressure wound dressing Autograft	S	Positive blood culture Streptococcus A	*Streptococcus pyogenes*
Krebs et al.^37^	1	P	F	Left leg	Hyperemia and edema of left leg	Site of venipuncture and extravasation	Ceftazidim Amikacin Debridement Amputation	D	Blood culture *E. coli* and *Morganella morgani*	*E. coli*
Lee et al.^38^	9		F	Buttock	Fever Inflammatory lesion on buttock		Ampicillin sulbactam Cefotaxime Vancomycin Debridement and drainage	S	DIC	MRSA
Lodha et al.^39^	19	T	M	Abdomen	Irritability Abdominal distension Temperature instability	Appendectomy	Penicillin Clindamycin Gentamicin	D	Shock	Coagulase‐negative *Staphylococcus*
Sharma et al.^40^	7	T	M	Neck	Fever Irritability Swelling and discoloration of neck	Insect bite	Cefotaxime Clindamycin Cloxacillin Debridement	S		Gram‐positive cocci
William et al.^41^	11	P	M	Neck	Lethargy Recurrent apnea Poor feeding		Augmentin Meropenem Vancomycin Colistin Amphotericin Debridement	S	Blood culture Enterococci	Mucor mycosis *Lichtheimia ramose*
Dhawan et al.^42^	9	T		scalp	Fever Erythema of scalp and face		Vancomycin Meropenem Clindamycin Colistin Debridement Skin graft	D	Multiorgan failure Septic shock	*Pseudomonas* *Acinetobacter baumannii*
Dhawan et al.^42^	21	T		scalp	Fever Irritability Puss discharge in occipital region	Head shaving	Meropenem debridement	S	CSF culture *Acinetobacter baumannii*	*Enterobacter cloacae*
Mercier et al.^43^	12	P	F	Chest	Chest erythema and discharge		Cefazolin Clindamycin		Blood culture MSSA	MSSA
Davey et al.^44^	1		F	Scalp		Fetal scalp monitoring Maternal blood and vaginal swab culture: Group A streptococcus	Meropenem Clindamycin Penicillin G IVIG 2 allografts 1 autograft	S	Acute renal failure NEC	Group A Streptococcus
Ozturk et al.^45^	22		F	Scalp			Skin autograft		Renal insufficiency Sepsis	Group A beta hemolytic Streptococcus
Siddiqi et al.^46^	5	T	M	scalp	Fever Lethargy Erythematous swelling scalp	Fetal scalp monitoring	Penicillin G Methicillin Kanamycin Skin autograft	S		*Peptostreptococcus anaerobius*, *Peptococcus asaccharolyticus*, and *Bacteroides melaninogenicus* *E. coli*
Siddiqi et al.^46^	3	T	M	scalp	Pale Erythema, warm, tender lesion on scalp	Fetal scalp monitoring	Penicillin G Methicillin Kanamycin	S	Cardiac arrest	*Fusobacterium* and Corynebacterium vaginale, oralis, melaninogenicus, and Peptococcus asaccharolyticus
Obu et al.^47^	21	T	F	Chest and abdomen	Ulcer over chest and abdomen Fever Poor feeding jaundice		Ceftriaxone Clindamycin Amikacin Metronidazole	S		*Staphylococcus aureus*
Obu et al.^47^	19	T	M	Arm Chest	Swelling left arm Fever Respiratory distress		Clindamycin Metronidazole Ceftazidime Imipenem Debridement Wound dressing with saline and honey	S		Proteus
Malik et al.^48^	10	T	F	Neck	Fever Redness of the neck		IV antibiotic Debridement Skin graft	S	Positive blood culture Osteomyelitis	MRSA
Bayileyegn et al.^49^	8	T	M	Scrotum	Fever Irritability Scrotal swelling		Ceftriaxone Metronidazole Debridement	S		*E. coli* *Staphylococcus aureus*
De La Torre et al.^50^	24	T	M	Scrotum	Fever Painful erythema on the groin		Cefotaxime Clindamycin Cloxacillin Debridement	S	Positive blood culture	*Streptococcus pyogenes*
Sawardekar^51^	7	T	F	Back	Hypothermia Bradycardia Multiple lesions	Insect and ant bite	Broad spectrum antibiotic Debridement Liposomal amphotericin B	D	Seizure	*Streptococcus agalactiae* Mucormycosis
Woodside^52^		T	M	Penis and Scrotum	Lethargy Hypothermia Penile and scrotal swelling	Plastibell circumcision	Nafcillin Kanamycin Penicillin Chloramphenicol Debridement Fasciotomy	S	Positive blood culture Micrococcus	Coagulase‐positive *Staphylococcus aureus*, *S epidermidis*, diphtheroids, nonhemolytic Streptococcus, and a‐hemolytic Streptococcus. *Clostridium perfringens*
Monu et al.^53^	19		M	Scrotum Abdomen	Phimosis Generalized pustular rash Fever Redness of anterior abdomen	Unsterile cord management	Gentamicin Cloxacillin solcoseryl dressing	S		
Monu et al.^53^	2	P	M	Abdomen	Abdominal distension pallor edema of the anterior abdominal wall and umbilicus	Unsterile cord management	Gentamicin Cloxacillin Metronidazole	D	Seizure	
Monu et al.^53^	5	T	F	Abdomen	Abdominal distension Dirty umbilical stump	Unsterile cord management	Gentamicin Cloxacillin Metronidazole	D	Septic shock	
Zgraj et al.^54^	27	T	M	Scrotum	Left scrotal swelling and edema Pyrexia Pussy discharge		amoxicillin with clavulanic acid and flucloxacillin Debridement	S		Enterobacter MSSA
Awe et al.^55^	7		M	Chest Abdomen	Swelling and discoloration of chest and abdomen Fever	Umbilical massage with hot water at home	Imipenem Metronidazole Gentamicin regular dressing with 1% povidone‐iodine and diluted honey	S	Positive blood culture	Coagulase‐negative *Staphylococcus aureus*
Kosloske et al.^56^	9	T	F	Abdomen	Umbilical discharge Erythema of abdomen		Ampicillin Gentamicin Clindamycin	S	Supraventricular tachycardia	Staphylococcus species (coagulase‐negative), diphtheroid, Clostridium sordellii, and Bacteroides fragilis
Ibekwe et al.^57^	21	T	F	Scalp	Scalp lesion Fever Pallor	Mother: HIV Rupture of membrane for 4 days Fever	Ceftriaxone Gentamycin Ciprofloxacin Debridement	S	Positive blood culture Staphylococcus aureus	B‐Haemolytic Streptococcus Klebsiella
Datta et al.^58^	10	T	F	Back	Fever Ulcer over back		Vancomycin Amikacin Debridement	S		*Staphylococcus aureus*
Tuncer et al.^59^	23	T	M	Shoulder and back	Fever Irritability Bruise on scalp	Varicella in two siblings	Vancomycin Imipenem Debridement Skin graft		Blood Staphylococcus warneri	*Staphylococcus aureus*
Chen et al.^60^	13		M	Upper limb	Progressive redness of upper limb		IV antibiotic Debridement Gentamicin dressing	S		
Wu et al.^17^	5		M	Back	Discoloration of back Fever Lethargy		Antibiotic Debridement	S	Positive blood culture	MSSA
Nnadozie et al.^61^	19	T	F	Ear	Ear sore	Ear piercing	Erythromycin Metronidazole	S		*S. aureus* *Pseudomonas*
Sakata et al.^62^	4		F		Fever Irritability Induration over the back and buttocks	Vaccination	Vancomycin Flucloxacillin Tazocin Acticoat 7 (nanocrystalline silver dressing) Vacuum‐Assisted Closure Skin graft	S		*S. aureus*
Scheffler et al.^63^	25	T		Scalp	Redness and swelling of the scalp		Antibiotic Debridement Skin graft	S		*Streptococcus pyogenes*
Scheffler et al.^63^	11	T		Scalp Forearm Leg	Necrotic tissue		Antibiotic Debridement Skin graft	S		*Streptococcus pyogenes*
Scheffler et al.^64^	15	P	M	Hand	Hand discoloration	IV site	Amphotericin B Debridement	S	Progression to forearm amputation	Zygomycosis
Chao et al.^24^	13	T	F	Back	Fever Erythema on back		Antibiotic Debridement Skin graft	S	Positive blood culture	*S. aureus*
Chao et al.^24^	17	T	M	Back	Fever Erythema on back		Antibiotic Debridement	S		*S. aureus*
Viel‐Thériault et al.^65^	5	P	M	Trunk shoulder	Bruising	PROM Chorioamnionitis	Vancomycin Cefotaxime Fluconazole	D	Septic shock	*Bacillus cereus* *S. aureus*
Presented case	10	P	F	Abdomen	Erythema		Vancomycin Meropenem Metronidazole	D	Sepsis	Nothing

Abbreviations: AML, Acute myeloid leukemia; BCG, Bacillus Calmette Guerin; D, death; DIC, Disseminated intravascular coagulation; F, female; GBS, Group B streptococcus; M, male; MRSA, Methicillin‐resistant *Staphylococcus aureus*; MSSA, Methicillin‐sensitive *Staphylococcus aureus*; NEC, Necrotizing enterocolitis; P, Preterm; PROM, premature rupture of membrane; *S. agalactiae*, *Streptococcus agalactiae*; S, survive; T, term.

In our literature review, together with our case, out of 77 neonates included, 38 (48.1%) were male and 32 (40.5%) females. Forty‐four neonates (55.7%) were born at term GA (GA≥37 weeks), while 14 (17.7%) were preterm (GA < 37), and in the others, GA was not mentioned. The mean age of presentation was 13.06 ± 8.66 days old.

The site of involvement was reported in 75 cases. Back was the most frequent site of involvement. In a pediatric systematic review, the lower extremity was reported as the most frequently involved site.^5^ Hsieh et al. in a neonatal review article reported the abdomen as the most frequent site of involvement and omphalitis as the most associated factor.^30^ The other sites are shown in Table 2.

**TABLE 2:** The site of involvement of necrotizing fasciitis in neonates.

Site of involvement	*N* (%)
Back	21 (26.6%)
Trunk (abdomen, chest)	19 (24.1%)
Perianal, penis, scrotum	15 (19.2%)
Scalp, ear, nasolacrimal	11 (14.1%)
Upper extremities	4 (5.1%)
Lower extremities	3 (3.8%)
Neck	3 (3.8%)
More than one site	2 (2.6%)

**FIGURE 2:**
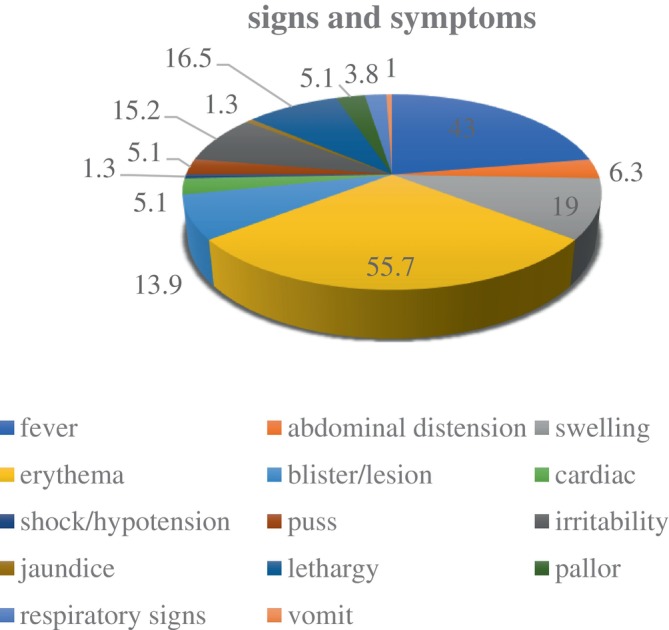
Clinical signs and symptoms.

The most frequent clinical presentations were erythema, fever, swelling, lethargy, irritability, and blister or lesion (Figure 2).

Among all the neonates in our review, the underlying cause was found in 36 neonates. Dirty cord management and fetal scalp monitoring were the most frequent causes (each one in four neonates). Intravenous line complication, circumcision, and NEC each had occurred in three neonates. The others were varicella in siblings, mother's pregnancy complications (PROM, HIV, GBS), environmental factors (lying in the park, insect bite), human interventions (rectal thermometer, head shaving, piercing, surgery, and vaccination), and immunodeficiency (AML, chemotherapy, LADS). In our case, we did not find any underlying cause.

To diagnose NF, we should consider it in patients with soft tissue infection (redness, swelling, warmth) and signs of systemic illness (fever, instability) along with crepitus and rapid progression. The diagnosis is confirmed through surgical exploration in the operating room, involving a thorough examination of the skin, subcutaneous tissue, fascial planes, and muscle.^66^

Polymicrobial infections are the cause of most NFs. The major cause of monomicrobial agents is reported *Streptococcus pyogenes* (group A streptococcus), a gram‐positive bacteria.^67^ In the pediatrics review, Streptococcus and Staphylococcus were the most common pathogens.^5^ In our neonatal literature review, staphylococcus aureus was the most monomicrobial agent, and *Streptococcus pyogenes* was reported in four cases (5.1%). Pandey et al. also reported *Staphylococcus aureus* as the most prevalent microbial agent.^68^ Table 3 shows the prevalence of organisms based on our review among neonates.

**TABLE 3:** The most common microorganisms that cause NF in neonates.

	Frequency, *N* (%)	Most prevalent, *N* (%)
Gram positive	50 (63.29%)	*Staphylococcus aureus*, 36 (45.6%)
Gram negative	17 (21.5%)	*Pseudomonas*, 5 (6.32%)
Fungal infection	5 (5%)	Mucormycosis, 5 (5%)

Fungal necrotizing fasciitis can occur in different situations, especially after intramuscular injection and traumatic wounds.^69^ It is not as prevalent as bacterial agents. In a pediatric review, it was responsible just for one infant.^5^ We should consider fungal infections, especially when recovery is not achieved by antimicrobial agents.^69^

The management of NF involves prompt and thorough surgical exploration and removal of necrotic tissues, along with the use of broad‐spectrum empirical antibiotics and the provision of hemodynamic support. Administrating antibiotic treatment without performing debridement is linked to a nearly 100% mortality rate.^70^ Hyperbaric oxygen therapy has received much attention recently.^71^ In this procedure, the patient receives 100% oxygen for a specified time and at a determined pressure. Hyperbaric oxygen therapy for necrotizing fasciitis is controversial, but survival and successful treatment were achieved in some studies.^72^, ^73^ Negative pressure wound healing is another procedure in which healing occurs by reduced edema, infection, and increased blood flow.^74^ In our review of the neonates, antibiotic therapy, debridement, skin graft, hyperbaric oxygen, IVIG, and GCSF were used. Table 4 shows the uncommon treatments and mortality based on our review.

**TABLE 4:** The uncommon treatments for NF in neonates.

Treatment	The number of patients received	Mortality
GCSF	1	1
IVIG	2	0
Negative pressure	5	1
Hyperbaric oxygen	1	0
Graft	16	2

In our literature review among neonates, vancomycin was the most widely used antibiotic. The other frequently used antibiotics are clindamycin, gentamycin, metronidazole, and meropenem, respectively. In most of the patients, a combination of antibiotics for covering gram‐positive, gram‐negative, and anaerobes were used.

There were seven kinds of dressing used in neonates including honey, silver, solcoseryl, Alginate, gentamycin, Mepitel and Acticoat, and iodine. The most prevalent one was honey with a successful outcome. All the neonates for whom honey dressing was used survived (four neonates). Honey was mentioned as a debriding agent with antimicrobial effects in some literature for NF.^75^

The mortality rate was 24.1% in our review. Gangopadhyay et al. reported 20% mortality in their study of 15 neonates.^76^ In another report, the mortality rate was 18.2% among 11 neonates.^68^ In the review article of Hsieh et al., 39 out of 69 neonates died (56.5%).^30^ In pediatric cases, it was reported 15.4%.^5^

## 4 CONCLUSION

Although necrotizing fasciitis is fatal and life‐threatening, by early recognition and appropriate treatment, mortality and morbidity rates are decreased. NF is suspected based on clinical signs, including erythema, warmth, and edema, often accompanied by systemic symptoms. Rapid progression is a hallmark feature of this condition. Surgical exploration is essential for both confirming the diagnosis and providing the treatment. Antibiotic therapy and debridement are the most frequently used treatment. Hyperbaric oxygen and negative pressure wound healing are effective treatments in some cases and need further investigation. Further studies are needed in neonates to investigate the risk factors, manifestations, and treatments of NF as there is limited research on innovative treatments in this population.

## AUTHOR CONTRIBUTIONS

**Roya Oboodi:** Conceptualization; supervision; writing – original draft; writing – review and editing. **Hamide Barzegar:** Conceptualization; data curation; supervision; writing – original draft; writing – review and editing. **Roozbeh Behzadi:** Conceptualization; data curation; writing – original draft; writing – review and editing.

## FUNDING INFORMATION

This case report received no funding.

## CONFLICT OF INTEREST STATEMENT

The authors declare that they have no competing interests.

## ETHICAL APPROVAL AND CONSENT TO PARTICIPATE

The publication of this case was approved by the Ethics committee of Shiraz University of Medical Sciences. Written informed consent was obtained from the patient's legal guardian for the publication of this case report and any accompanying images. A copy of the written consent is available for review by the Editor‐in‐Chief of this journal. All methods were performed by the ethical standards as laid down in the Declaration of Helsinki and its later amendments or comparable ethical standards.

## CONSENT

Written informed consent was obtained from the patient's legal guardian for the publication of this case report and any accompanying images.

## Data Availability

Materials and data provided in this case study are available from the corresponding author on reasonable request.

## References

[1] Chen LL , Fasolka B , Treacy C . Necrotizing fasciitis: a comprehensive review. Nursing. 2020;50(9):34‐40. 10.1097/01.NURSE.0000694752.85118.62 PMC8828282 32826674

[2] Pfeifle VA , Gros SJ , Holland‐Cunz S , Kämpfen A . Necrotizing fasciitis in children due to minor lesions. J Pediatr Surg Case Rep. 2017;25:52‐55.

[3] Waldhausen JH , Holterman MJ , Sawin RS . Surgical implications of necrotizing fasciitis in children with chickenpox. J Pediatr Surg. 1996;31(8):1138‐1141. 8863250 10.1016/s0022-3468(96)90103-7

[4] VanderMeulen H , Pernica JM , Roy M , Kam AJ . A 10‐year review of necrotizing fasciitis in the pediatric population: delays to diagnosis and management. Clin Pediatr. 2017;56(7):627‐633. 10.1177/0009922816667314 27663964

[5] Zundel S , Lemaréchal A , Kaiser P , Szavay P . Diagnosis and treatment of pediatric necrotizing fasciitis: a systematic review of the literature. Eur J Pediatr Surg. 2017;27(2):127‐137. 27380058 10.1055/s-0036-1584531

[6] Ameh EA , Awotula OP , Amoah JN . Spontaneous scrotal faecal fistula in infants. Pediatr Surg Int. 2002;18(5):524‐525. 12415400 10.1007/s00383-002-0754-y

[7] Kuroda J , Inoue N , Satoh H , Fukuzawa R , Terakawa T , Hasegawa Y . Neonatal necrotizing fasciitis of the scrotum caused by S treptococcus agalactiae. Pediatr Int. 2015;57(2):e56‐e58. 25712264 10.1111/ped.12563

[8] Barker L , Pringle K , Cusack J . Necrotising fasciitis with *Escherichia coli* in a newborn infant after abdominal surgery. Arch Dis Child Fetal Neonatal ed. 2013;98(5):F404. 23572343 10.1136/archdischild-2013-303771

[9] Inoue S , Odaka A , Hashimoto D , et al. Rare case of disseminated neonatal zygomycosis mimicking necrotizing enterocolitis with necrotizing fasciitis. J Pediatr Surg. 2011;46(10):e29‐e32. 10.1016/j.jpedsurg.2011.06.018 22008359

[10] Lebel E , Karasik M , Shahroor‐Karni S , Peyser A . Necrotizing upper limb fasciitis in a newborn: an uncommon life‐threatening event. J Pediatr Orthop B. 2012;21(6):536‐538. 22080297 10.1097/BPB.0b013e32834dd206

[11] Melchionda F , Pession A . Negative pressure treatment for necrotizing fasciitis after chemotherapy. Pediatr Rep. 2011;3(4):e33. 22355518 10.4081/pr.2011.e33 PMC3283201

[12] Thapa R , Mallick D , Biswas B . Necrotizing fasciitis following BCG vaccination. Indian Pediatr. 2011;48(3):235‐237. 21478557

[13] Walls T , Williams G , Adams S , Sugo E , Mulcahy D . Neonatal necrotising fasciitis following superficial skin infection with community‐associated methicillin‐resistant Staphylococcus aureus. J Paediatr Child Health. 2010;47(12):918‐920. 20604827 10.1111/j.1440-1754.2010.01732.x

[14] Shi S , Jia S , Liu J , Chen G , He S . Continuous renal replacement therapy as a supportive treatment for acute pediatric necrotizing fasciitis. Cell Biochem Biophys. 2014;69(2):219‐223. 24242188 10.1007/s12013-013-9785-3

[15] Peker E , Kirimi E , Tuncer O , Ceylan A , Cagan E , Dogan M . Necrotizing fasciitis caused by Staphylococcus epidermidis in a neonate with extremely low birthweight. J Dermatol. 2010;37(7):671‐673. 20629835 10.1111/j.1346-8138.2010.00840.x

[16] Özkan H , Kumtepe S , Turan A , Özkan S . Perianal necrotizing fasciitis in a neonate. Indian J Pediatr. 1997;64(1):116‐118. 10771824 10.1007/BF02795792

[17] Wu Y , Jiang X . Neonatal necrotising fasciitis. Arch Dis Child Fetal Neonatal ed. 2022;107(1):97. 33214153 10.1136/archdischild-2020-320818

[18] Negosanti L , Aceti A , Bianchi T , et al. Adapting a vacuum assisted closure dressing to challenging wounds: negative pressure treatment for perineal necrotizing fasciitis with rectal prolapse in a newborn affected by acute myeloid leukaemia. Eur J Dermatol. 2010;20(4):501‐503. 20406723 10.1684/ejd.2010.0964

[19] Narvey M , Byrne P , Fraser D . Necrotizing fasciitis of the Abdominal Wall in a premature infant: a case study. Neonatal Netw. 2017;36(1):26‐31. 28137350 10.1891/0730-0832.36.1.26

[20] Ganesh A , Al‐Zuhaibi SS , Bialasiewicz A , Ahmed S , Al‐Tamemi S , et al. Necrotizing pseudomonas infection of the ocular adnexa in an infant with leukocyte adhesion defect. J Pediatr Ophthalmol Strabismus. 2007;44(4):199‐200. 17694822 10.3928/01913913-20070701-09

[21] Lazzeri D , Lazzeri S , Figus M , Nardi M , Pantaloni M , Agostini T . Immunocompromise as major risk factor for necrotising infections of orbital and ocular adnexa caused by Pseudomonas aeruginosa. Orbit. 2010;29(6):373‐376. 20954843 10.3109/01676830.2010.509533

[22] Bliss DP Jr , Healey PJ , Waldhausen JH . Necrotizing fasciitis after Plastibell circumcision. J Pediatr. 1997;131(3):459‐462. 9329429 10.1016/s0022-3476(97)80078-9

[23] Bodemer C , Panhans A , Chretien‐Marquet B , Cloup M , Pellerin D , de Prost Y . Staphylococcal necrotizing fasciitis in the mammary region in childhood: a report of five cases. J Pediatr. 1997;131(3):466‐469. 9329431 10.1016/s0022-3476(97)80080-7

[24] Chao H‐C , Kong M‐S , Lin T‐Y . Diagnosis of necrotizing fasciitis in children. J Ultrasound Med. 1999;18(4):277‐281. 10206215 10.7863/jum.1999.18.4.277

[25] Christensen R , Pysher T , Christensen S . Case report: perianal necrotizing fasciitis in a near‐term neonate. J Perinatol. 2007;27(6):390‐391. 17522688 10.1038/sj.jp.7211733

[26] Dehority W , Wang E , Vernon PS , Lee C , Perdreau‐Remington F , Bradley J . Community‐associated methicillin‐resistant Staphylococcus aureus necrotizing fasciitis in a neonate. Pediatr Infect Dis J. 2006;25(11):1080‐1081. 17072137 10.1097/01.inf.0000243158.25713.29

[27] Ekingen G , Isken T , Agir H , Öncel S , Günlemez A . Fournier's gangrene in childhood: a report of 3 infant patients. J Pediatr Surg. 2008;43(12):e39‐e42. 10.1016/j.jpedsurg.2008.09.014 19040919

[28] Fustes‐Morales A , Gutierrez‐Castrellon P , Duran‐Mckinster C , Orozco‐Covarrubias L , Tamayo‐Sanchez L , Ruiz‐Maldonado R . Necrotizing fasciitis: report of 39 pediatric cases. Arch Dermatol. 2002;138(7):893‐899. 12071816 10.1001/archderm.138.7.893

[29] Hayani KC , Mathew R , Oyedele T , Hulten KG . Neonatal necrotizing fasciitis due to community‐acquired methicillin resistant Staphylococcus aureus. Pediatr Infect Dis J. 2008;27(5):480‐481. 18382381 10.1097/INF.0b013e31816bceb0

[30] Hsieh W‐S , Yang P‐H , Chao H‐C , Lai J‐Y . Neonatal necrotizing fasciitis: a report of three cases and review of the literature. Pediatrics. 1999;103(4):e53. 10103345 10.1542/peds.103.4.e53

[31] Ignacio RC , Falcone RA Jr , Warner BW . Necrotizing fasciitis: a rare complication of neonatal necrotizing enterocolitis. J Pediatr Surg. 2005;40(11):1805‐1807. 16291176 10.1016/j.jpedsurg.2005.07.003

[32] Zuloaga‐Salcedo S , Contreras‐Ruiz J , Dominguez‐Cherit J , Vega‐Memije E . An approach to the management of necrotising fasciitis in neonates. Int Wound J. 2005;2(2):178‐180. 16722868 10.1111/j.1742-4801.2005.00104.x PMC7951346

[33] Orii K , Iwao Y , Higuchi W , Takano T , Yamamoto T . Molecular characterization of methicillin‐resistant Staphylococcus aureus from a fatal case of necrotizing fasciitis in an extremely low‐birth‐weight infant. Clin Microbiol Infect. 2010;16(3):289‐292. 19519846 10.1111/j.1469-0691.2009.02806.x

[34] Dunlop RL , Eadie P . Idiopathic neonatal necrotising fasciitis caused by community‐acquired MSSA encoding Panton valentine Leukocidin genes. J Plast Reconstr Aesthet Surg. 2011;64(11):1522‐1524. 21511548 10.1016/j.bjps.2011.03.041

[35] Casey DM , Stebbins K , Howland V . Necrotizing fasciitis: a case report of a premature infant with necrotizing enterocolitis. J Pediatr Nurs. 2013;28(5):486‐491. 23276506 10.1016/j.pedn.2012.12.001

[36] Kimia R , Azoury SC , Allukian M III , Nguyen PD . Cultured epidermal autograft for Total scalp reconstruction in a neonate following necrotizing fasciitis. Ann Plast Surg. 2020;85(3):276‐280. 31923018 10.1097/SAP.0000000000002235

[37] Krebs VLJ , Koga KM , Diniz EMA , Ceccon MEJ , Vaz FAC . Necrotizing fasciitis in a newborn infant: a case report. Rev Hosp Clin Fac Med Sao Paulo. 2001;56:59‐62. 11460206 10.1590/s0041-87812001000200005

[38] Lee K‐H , Hahn W‐H , Park S‐S , Cho B‐S , Kim S‐D . Necrotizing fasciitis in a neonate—the role of keratinocyte allografts. Neonatology. 2009;96(1):19‐22. 19202344 10.1159/000200166

[39] Lodha A , Wales PW , James A , Smith CR , Langer JC . Acute appendicitis with fulminant necrotizing fasciitis in a neonate. J Pediatr Surg. 2003;38(11):E5‐E6. 10.1016/j.jpedsurg.2003.08.014 14614733

[40] Sharma V , Panda NK , Kapoor A , Angrish P , Raj RR . Necrotising fasciitis of neck in a 7 day neonate following insect bite. Indian journal of otolaryngology and head & neck. Surgery. 2021;74:1‐5. 10.1007/s12070-021-02421-2 PMC9895229 36742649

[41] William A , Kaur R , Rawat D , Kandir NS , Sharma A . Necrotizing fasciitis in neonate by Lichtheimia ramosa: a case study. Access. Microbiology. 2022;4(3):1‐6. 10.1099/acmi.0.000327 PMC9175980 35693464

[42] Dhawan SR , Vaidya PC , John JJ , et al. Necrotizing fasciitis of scalp and neck in neonates. APSP J Case Rep. 2017;8(3):23. 28540194 10.21699/ajcr.v8i3.554 PMC5423892

[43] Mercier G , Parrado RH , Jenkins D , Streck C . Use of a dermal matrix for an open chest wound in a newborn with complicated necrotizing fasciitis. Am Surg. 2021;89:2767‐2769. 34730444 10.1177/00031348211054549

[44] Davey C , Moore AM . Necrotizing fasciitis of the scalp in a newborn. Obstet Gynecol. 2006;107(2 Part 2):461‐463. 16449149 10.1097/01.AOG.0000164094.02571.77

[45] Ozturk S , Zor F , Karslioglu Y , Sengezer M . Idiopathic neonatal necrotizing fasciitis of the scalp: a case report. Eur J Plast Surg. 2005;28(5):368‐370.

[46] Siddiqi SF , Taylor PM . Necrotizing fasciitis of the scalp: a complication of fetal monitoring. Am J Dis Child. 1982;136(3):226‐228. 7064948 10.1001/archpedi.1982.03970390040013

[47] Obu H , Obumneme‐Anyim I , Iloh K , Akubuilo U , Okwesili O , Achebe U . Neonatal necrotizing fasciitis: two case reports and literature review. JHAD. 2020;25(2):144.

[48] Malik R , Saluja S , Modi M , et al. Scalp necrotizing fasciitis—a case report. J Neonatol. 2021;35(1):42‐44.

[49] Bayileyegn NS , Tareke AA . Fournier's gangrene in an eight‐day‐old male neonate, a case report. Int J Surg Case Rep. 2022;94:106982. 35405509 10.1016/j.ijscr.2022.106982 PMC9010749

[50] De La Torre M , Solé C , Fanjul M , et al. Neonatal Fournier's gangrene: avoiding extensive debridement. Pediatr Infect Dis J. 2021;40(10):e384‐e387. 34292272 10.1097/INF.0000000000003224

[51] Sawardekar KP . Gangrenous necrotizing cutaneous mucormycosis in an immunocompetent neonate: a case report from Oman. J Trop Pediatr. 2018;64(6):548‐552. 29253258 10.1093/tropej/fmx094

[52] Woodside JR . Necrotizing fasciitis after neonatal circumcision. Am J Dis Child. 1980;134(3):301‐302. 6444778 10.1001/archpedi.1980.02130150055015

[53] Monu J , Okolo A . Neonatal necrotizing fasciitis—a complication of poor cord hygiene: report of three cases. Ann Trop Paediatr. 1990;10(3):299‐303. 1703748 10.1080/02724936.1990.11747446

[54] Zgraj O , Paran S , O'Sullivan M , Quinn F . Neonatal scrotal wall necrotizing fasciitis (Fournier gangrene): a case report. J Med Case Reports. 2011;5(1):1‐3. 10.1186/1752-1947-5-576 PMC3264539 22151925

[55] Awe OO , Kesieme EB , Kayode‐Adedeji B , Aigbonoga QO . Necrotizing fasciitis of the chest in a neonate in southern Nigeria. Case reports. Pediatrics. 2014;2014:1‐3. 10.1155/2014/818059 PMC4293782 25610690

[56] Kosloske AM , Bartow SA . Debridement of periumbilical necrotizing fasciitis: importance of excision of the umbilical vessels and urachal remnant. J Pediatr Surg. 1991;26(7):808‐810. 1895189 10.1016/0022-3468(91)90144-i

[57] Ibekwe M , Ojukwu J , Ibekwe R . Unusual presentation of necrotizing fasciitis in an HIV exposed infant: a case report. Niger J Paediatr. 2011;38(3):142‐145.

[58] Datta S , Bit UK , Maity S . A case of necrotizing fasciitis in a neonate. The Child and Newborn. 2010;34:34‐36.

[59] Tuncer O , Ataş B , Kırımi E , Akbayram S , Atik B , Çaksen H . Acute necrotizing fasciitis developed due to staphylococcal infection during neonatal varicella. J Pediatr Infect Dis. 2006;1(4):235‐237.

[60] Chen J , Shen W , Cui J . Iatrogenic necrotizing fasciitis in a neonate. J Craniofac Surg. 2011;22(5):1985‐1986. 21959495 10.1097/SCS.0b013e31822eabe7

[61] Nnadozie UU , Ezeanosike OB , Maduba CC , Obu DC , Unigwe USD . Necrotizing soft tissue infection of both ear lobules occurring concomitantly in a set of twins following non‐aseptic ear piercing: a case report. BMC Pediatr. 2020;20(1):54. 32020856 10.1186/s12887-020-1952-2 PMC7001364

[62] Sakata S , Das Gupta R , Leditschke JF , Kimble RM . Extensive necrotising fasciitis in a 4‐day‐old neonate: a successful outcome from modern dressings, intensive care and early surgical intervention. Pediatr Surg Int. 2009;25(1):117‐119. 18982332 10.1007/s00383-008-2289-3

[63] Kothari PR , Kulkarni B . Neonatal necrotizing fascitis. Indian Pediatr. 2004;41(10):1070‐1071. 15523143

[64] Scheffler E , Miller GG , Classen DA . Zygomycotic infection of the neonatal upper extremity. J Pediatr Surg. 2003;38(7):E16‐E17. 10.1016/s0022-3468(03)00215-x 12861594

[65] Viel‐Thériault I , Puthattayil ZB , Ferretti E . Fulminant necrotizing soft‐tissue infection in an extremely low gestational age infant. Pediatr Infect Dis J. 2021;40(5):e189‐e190. 33480664 10.1097/INF.0000000000003064

[66] Dennis L , Stevens M , Baddour LM . Necrotizing soft tissue infections. Uptodate. 2023.

[67] Tessier JM , Sanders J , Sartelli M , et al. Necrotizing soft tissue infections: a focused review of pathophysiology, diagnosis, operative management, antimicrobial therapy, and pediatrics. Surg Infect (Larchmt). 2020;21(2):81‐93. 31584343 10.1089/sur.2019.219

[68] Pandey V , Gangopadhyay A , Gupta D , Sharma S , Kumar V , Tiwari P . Neonatal necrotising fasciitis managed conservatively: an experience from a tertiary Centre. J Wound Care. 2014;23(5):270‐273. 24810311 10.12968/jowc.2014.23.5.270

[69] Chander J , Stchigel AM , Alastruey‐Izquierdo A , et al. Fungal necrotizing fasciitis, an emerging infectious disease caused by Apophysomyces (Mucorales). Rev Iberoam Micol. 2015;32(2):93‐98. 25576377 10.1016/j.riam.2014.06.005

[70] Anaya DA , Dellinger EP . Necrotizing soft‐tissue infection: diagnosis and management. Clin Infect Dis. 2012;2007:44‐710. 10.1086/511638 17278065

[71] Edlich RF , Cross CL , Dahlstrom JJ , Long WB III . Modern concepts of the diagnosis and treatment of necrotizing fasciitis. J Emerg Med. 2010;39(2):261‐265. 19081698 10.1016/j.jemermed.2008.06.024

[72] Memar MY , Yekani M , Alizadeh N , Baghi HB . Hyperbaric oxygen therapy: antimicrobial mechanisms and clinical application for infections. Biomed Pharmacother. 2019;109:440‐447. 30399579 10.1016/j.biopha.2018.10.142

[73] Soh CR , Pietrobon R , Freiberger JJ , et al. Hyperbaric oxygen therapy in necrotising soft tissue infections: a study of patients in the United States Nationwide inpatient sample. Intensive Care Med. 2012;38(7):1143‐1151. 22527074 10.1007/s00134-012-2558-4

[74] Agarwal P , Kukrele R , Sharma D . Vacuum assisted closure (VAC)/negative pressure wound therapy (NPWT) for difficult wounds: a review. J Clin Orthop Trauma. 2019;10(5):845‐848. 31528055 10.1016/j.jcot.2019.06.015 PMC6739293

[75] Dogra RS , Chaudhary R , Poonam VD , Thakur A , Bhatia R . Honey is an ideal biological agent in the Management of Necrotizing Fasciitis: a case report. AIMDR. 2020;6(4):18.

[76] Gangopadhyay AN , Pandey A , Upadhyay VD , Sharma SP , Gupta DK , Kumar V . Neonatal necrotising fasciitis–Varanasi experience. Int Wound J. 2008;5(1):108‐112. 18081783 10.1111/j.1742-481X.2007.00350.x PMC7951785

